# Estimation of salt intake assessed by urinary excretion of sodium over 24 h in Spanish subjects aged 7–11 years

**DOI:** 10.1007/s00394-015-1067-y

**Published:** 2015-10-19

**Authors:** A. Aparicio, E. Rodríguez-Rodríguez, E. Cuadrado-Soto, B. Navia, A. M. López-Sobaler, R. M. Ortega

**Affiliations:** 1 Departamento de Nutrición Y Bromatología I (Nutrición), Facultad de Farmacia, Universidad Complutense de Madrid, Madrid, Spain; 2 Sección Departamental de Química Analítica, Facultad de Farmacia, Universidad Complutense de Madrid, Madrid, Spain; 3 UCM Research Group: VALORNUT-920030, Madrid, Spain

**Keywords:** Urinary sodium excretion, Salt intake, Schoolchildren, Spain

## Abstract

**Purpose:**

High intake of salt is associated with early development of cardiovascular risk factors (e.g., hypertension, obesity). In “developed” countries, individuals frequently exceed dietary recommendations for salt intake. Taking into account the limited data on sodium intake by 24-h excretion in urine in schoolchildren, we wished to determine baseline salt intake in Spanish subjects aged 7–11 years.

**Methods:**

The present study was an observational study involving 205 schoolchildren (109 boys and 96 girls) selected from various Spanish provinces. Sodium intake was ascertained by measuring sodium excretion in urine over 24 h. Creatinine was used to validate completeness of urine collections. The correlation between fat-free mass determined by anthropometry and that determined via urinary excretion of creatinine was calculated (*r* = 0.651; *p* < 0.001).

**Results:**

Mean 24-h urinary excretion of sodium was 132.7 ± 51.4 mmol/24 h (salt equivalent: 7.8 ± 3.1 g/day). Hence, 84.5 % of subjects aged ≤10 years had intakes of >4 g salt/day, and 66.7 % of those aged >10 years had intakes of >5 g salt/day. Urinary excretion of sodium was correlated with systolic blood pressure and diastolic blood pressure (*r* = 0.1574 and *r* = 0.1400, respectively). Logistic regression analyses, adjusted by sex, showed that a high body mass index (odds ratio = 1.159; 95 % CI 1.041–1.290; *p* < 0.05) was associated with an increased likelihood of high urinary excretion of sodium.

**Conclusions:**

Sodium intake, as estimated by 24-h urinary excretion, was (on average) higher than recommended. Reducing the sodium content children’s diet is a sound policy to reduce cardiovascular risk.

## Introduction

A high intake of salt is associated with the risk of early development of cardiovascular risk factors such as hypertension [1, 2]. Subjects with high blood pressure (BP) at an early age are more likely to develop hypertension in the future [3]. Simultaneously, high intakes of salt increase the risk of other disorders, such as kidney disease, osteoporosis, the formation of kidney stones, and stomach cancer (of which it is thought to be the main cause) [1, 4–6].

In “developed” countries, individuals frequently exceed dietary recommendations for salt [7–10]. Disorders that result from such behavior can be important public health problems and involve considerable socioeconomic costs [10, 11]. Reducing salt intake is one of the easiest, most efficient, and cost-effective ways to control this problem [1, 12]. In Spain, the Ministry of Health is developing a strategy to reduce the salt intake of the population according to recommendations set by the World Health Organization (WHO)/Food and Agriculture Organization of the United Nations (5 g/day) [13].

The best method of estimating sodium intake is measurement of its excretion over 24 h [14]. Use of dietary surveys and databases detailing food composition can underestimate sodium intake [8, 15].

There are important logistical difficulties in collecting complete 24-h urine samples, particularly in children. In Spain, only two studies have measured 24-h urinary excretion of sodium [16, 17]. We wished to determine baseline salt intake in Spanish subjects aged 7–11 years.

## Subjects and methods

The study protocol was approved by the Ethics Committee for Clinic Review of Clinic San Carlos Hospital, which is part of the Complutense University of Madrid (Madrid, Spain).

### Recruitment of subjects

This observational study was conducted between February and June 2014. It involved five primary schools from various Spanish provinces, including the capital city of each province and a semi-urban/rural city. All schools were chosen randomly. Of the 10 schools contacted by telephone, five accepted the invitation to be included in the study. Permission was requested to meet the parents of children in the age group 7–11 years. Once permission was given, the details of the study were explained to parents and all questions answered. Written informed consent was then sought to include their children in the study.

A total of 1232 children were given the opportunity to participate in the study. Of these, 275 children provided written informed consent and 70 were excluded. The final sample, therefore, comprised 205 participants aged 7–11 years. For these subjects, complete information was available regarding health problems, anthropometric data, and 24-h urine samples. All subjects took part voluntarily.

The exclusion criteria were: (1) lack of authorization to take part or non-acceptance of any of the conditions required for the study to proceed; (2) non-attendance on days when tests or interviews were conducted; (3) having a disease that might affect the results (e.g., diabetes mellitus, hypertension, renal disease), altered food habits (and, therefore, nutrient intake), or would warrant non-inclusion. Also, those urine samples with incomplete micturitions were not included in the study.

### Methods

#### Health variables

Information was collected from all participants on health problems as well as consumption of medications, supplements, and manufactured foods such as snacks and processed foods (ready meals, entrees, side dishes, etc.). BP was measured in the right arm of seated patients following a 5-min rest period using an HEM-907XL automated sphygmomanometer (Omron Health Care, Vernon Hills, IL, USA). BP was measured in triplicate with an interval between measures of at least 5 min. All readings were taken by a trained technician [18].

Schoolchildren were classified as “normotensive” and “hypertensive” according to the criteria for Spanish people set by Díaz et al. [19].

#### Anthropometric data

All measurements were taken at schools in the morning and in accordance with norms set out by the WHO [20].

Weight and height were determined using a digital electronic balance (range 0.1–150 kg; precision, 100 g; Alpha; Seca, Igni, France) and a digital stadiometer (70–205 cm; 1 mm; Harpenden Pfifter, Carlstadt, NJ, USA), respectively. For these measurements, children were barefoot and wore only underwear. The body mass index (BMI) was then calculated.

Overweight and obesity were defined according to BMI-specific percentiles for age and sex in the reference population according to criteria set by Fernández et al. [21].

Measurements for the waist and hip were determined using a flexible metallic tape (range 0–150 cm; precision, 1 mm; Holtain, Crymych, Wales). These were measured while the subject was standing relaxed and with the tape held snugly around the body (but not so tight that subcutaneous adipose tissue was compressed). The waist was measured midway between the inferior margin of the last rib and the crest of the ileum, in the horizontal plane. The maximum circumference encircling the buttocks and the pubic symphysis in the horizontal plane constituted hip circumference. An assistant helped to hold the tape on the side of the subject’s body opposite the measurer. The mean value of the three measurements was used for analyses. The waist–hip ratio was then calculated.

Thicknesses of bicep, tricep, subscapular, and suprailiac skinfolds were measured on the right side in triplicate to the nearest millimeter using a skinfold caliper (range 0–40 mm; precision, 0.1 mm; Holtain). All calipers were calibrated each day before taking measurements.

Percentage body fat (%BF) was determined using the equation derived by Deurenberg et al. [22]:

$$\begin{aligned} & {\text{Male}}\;{\text{individuals}}\;{\text{aged}}\;2{-}18\;{\text{years}}\;\left( {\% {\text{BF}}} \right) = \left[ {562-4.2 \, \left( {{\text{age}}-2} \right)} \right]/D-\left[ {525-4.7 \, \left( {{\text{age}}-2} \right)} \right], \hfill \\ & {\text{Female}}\;{\text{individuals}}\;{\text{aged}}\;2{-}10\;{\text{years}}\;\left( {\% {\text{BF}}} \right) = \left[ {562-1.1 \, \left( {{\text{age}}-2} \right)} \right]/D-\left[ {525-1.4 \, \left( {{\text{age}}-2} \right)} \right], \hfill \\ & {\text{Female}}\;{\text{individuals}}\;{\text{aged}}\;10{-}18\;{\text{years}}\;\left( {\% {\text{BF}}} \right) = \left[ {533-7.3 \, \left( {{\text{age}}-10} \right)} \right]/D-\left[ {514-8 \, \left( {{\text{age}}-10} \right)} \right], \hfill \\ \end{aligned}$$

where *D* was calculated according to age as follows:

$$\begin{aligned} & {\text{Male}}\;{\text{individuals:}}\;D = 1.1133{-}0.0561 \times \log \;\left( {\Sigma \;4\;{\text{skinfolds}}} \right) + 1.7 \, ( {{\text{age}}\;10^{ - 3} }) \\ & {\text{Female}}\;{\text{individuals:}}\;D = 1.1187{-}0.063 \times \log \;\left( {\Sigma \;4\;{\text{skinfolds}}} \right) + 1.9 \, ( {{\text{age}}\;10^{ - 3} } ) \\ \end{aligned}$$

Using these values and knowing the subject’s body weight, then fat mass and fat-free mass can be calculated:

$$\begin{aligned} & {\text{Fat}}\;{\text{mass}}\;\left( {\text{kg}} \right) = {\text{body}}\;{\text{fat}}\;\left( \% \right) \times {\text{body}}\;{\text{weight/}}100 \\ & {\text{Fat-free}}\;{\text{mass}}\;\left( {\text{kg}} \right) = {\text{body}}\;{\text{weight}}\;\left( {\text{kg}} \right)-{\text{fat}}\;{\text{mass}}\;\left( {\text{kg}} \right) \\ \end{aligned}$$

#### Urine testing

To ensure compliance in the 24-h urine collection, the children and their parents were carefully instructed in the collection procedure and also received written directions. Children were asked to void their bladders at 8 o’clock p.m.; this micturition was completely discarded, and the time was registered (start of collection). All the urine passed for the next 24 h was collected, including the complete sample produced at 8 o’clock p.m. the next day. This protocol has been adapted from Neubert et al. [23]. All micturitions were stored immediately in preservative-free, 1-L plastic containers at temperatures <−12 °C before transfer to the laboratory.

Volume, as well as the content of sodium, potassium, and creatinine, of 24-h urine was determined. Urinary levels of sodium and potassium were quantified using an indirect potentiometer with selective solid membranes for each ion connected to an AU 5400 Autoanalyzer (Olympus, Mishima, Japan): coefficient of variation (CV) = 1.0 % for sodium and 1.1 % for potassium [24].

Creatinine levels were determined according to a modification of the Jaffé reaction using the same apparatus. Color intensity was measured at 520 nm (CV = 2.8 %) [25]. All reagents were supplied by Olympus.

To confirm appropriate collection of 24-h urine, the correlation between urinary levels of creatinine and muscular mass of each subject was taken into account [26]. Fat-free mass was calculated bearing in mind the creatinine excreted over 24 h in urine using the following equation [27]:

$${\text{Fat-free}}\;{\text{mass}}\;\left( {\text{kg}} \right) = 0.02908 \times {\text{creatinine}}\;\left( {\text{mg/day}} \right) + 7.38$$

### Statistical analysis

Normality of all data was checked. The results for all variables are expressed as mean ± standard deviation (SD), medians with interquartile range or percentages where appropriate.

Differences regarding sex, BP, or BMI groups were tested using the Student’s *t* test (or the Mann–Whitney test if the distribution of results was not homogeneous). For qualitative variables, the *χ* ^2^ test was used.

Pearson’s linear correlation coefficient between FFM-A and FFM-F was calculated. Likewise, Pearson’s correlation coefficient was used to assess the association between 24-h urine sodium and both systolic blood pressure (SBP) and diastolic blood pressure (DBP). Relationships between 24-h urine sodium and SPB or DBP were examined by multiple regression analysis, were sex, age and BMI were included as covariates. Logistic regression analysis was used to explore the likelihood of high 24-h urine sodium (>100 mmol/24 h) and BMI considering sex as covariate, expressing the OR and the 95 % CI.

All calculations were made using SPSS v19.0 (IBM, Armonk, NY, USA). *p* < 0.05 was considered significant.

## Results

A total of 205 children (109 were male and 96 were female) aged 7–11 years (mean ± SD: 8.8 ± 1.2 years) completed the study. Personal, anthropometric, and blood-pressure data of participants are shown in Table 1.

**Table 1:** Personal and anthropometric characteristics of the study population according to sex

	Total		Boys		Girls	
	Mean ± SD	Median (interquartile range)	Mean ± SD	Median (interquartile range)	Mean ± SD	Median (interquartile range)
*N*	205		109		96	
Age (years)^a^	8.8 ± 1.2	9 (8–10)	8.8 ± 1.2	9 (8–10)	8.9 ± 1.2	9 (8–10)
Weight (kg)^a^	35.3 ± 8.3	34.0 (30.0–40.0)	35.0 ± 8.4	33.0 (29.0–40.0)	35.6 ± 8.3	35.5 (30.0–40.0)
Height (cm)	136.9 ± 8.6	137.2 (130.2–142.7)	137.0 ± 8.0	136.5 (130.8–142.4)	136.9 ± 9.4	137.9 (129.0–143.0)
BMI (kg/m^2^)^a^	18.6 ± 3.5	18.1 (16.3–20.4)	18.4 ± 3.8	17.6 (16.2–20.0)	18.8 ± 3.0	18.5 (16.7–20.6)
Overweight (%)	23.5		23.3		23.8	
Obese (%)	6.8		10.1*		2.9*	
Waist (cm)^a^	64.4 ± 8.5	62.9 (58.2–68.7)	64.6 ± 9.1	61.9 (58.3–67.8)	64.3 ± 7.9	64.0 (58.2–68.9)
Waist/height	0.49 ± 0.07	0.48 (0.44–0.53)	0.49 ± 0.08	0.48 (0.43–0.53)	0.49 ± 0.06	0.48 (0.44–0.53)
BF-A (%)	20.1 ± 6.8	19.9 (14.8–25.5)	18.0 ± 6.9***	16.1 (12.3–23.5)	22.4 ± 6.0***	22.1 (18.0–26.8)
BF-A (kg)^a^	7.5 ± 4.0	6.6 (4.6–9.9)	6.8 ± 4.1**	5.7 (3.4–9.3)	8.3 ± 3.7**	8.0 (5.5–10.7)
FFM-A (kg)	27.8 ± 5.0	27.2 (24.3–30.8)	28.3 ± 4.7	27.7 (24.9–31.2)	27.2 ± 5.3	26.8 (24.1–30.1)
FFM-F (kg)	28.3 ± 5.5	27.8 (24.4–32.1)	29.2 ± 5.5*	29.1 (25.6–32.8)	27.3 ± 5.5*	26.7 (23.4–31.1)
Blood pressure						
Systolic (mmHg)	100.6 ± 14.6	101.0 (93.0–109.0)	101.2 ± 16.8	102.0 (94.0–110.0)	99.9 ± 11.8	100.0 (92.8–107.0)
Diastolic (mmHg)	65.1 ± 11.4	64.5 (60.0–71.0)	64.9 ± 13.6	63.5 (60.0–71.0)	65.2 ± 8.1	64.5 (60.0–71.0)

*BMI* body mass index, *BF-A* body fat measured by anthropometry, *FFM-A* fat-free mass determined by anthropometry, *FFM-F* fat-free mass determined by formula [27]

* *p* < 0.05; ** *p* < 0.01; *** *p* < 0.001 (differences between boys and girls)

^a^Variable not normally distributed

Although for cross-sectional studies, no reasonable tool is available to identify incomplete 24-h urine samples, Remer et al. [28] indicated that a daily creatinine excretion rates that fall below 0.1 mmol/kg/day in healthy children are highly suspected to be incomplete. According to these criteria, none of the children studied had urinary creatinine excretion lower than 0.1 mmol/kg/day (range values: 0.1–0.3 mmol/kg/day).

Excretion of creatinine, sodium, and potassium was significantly higher in boys than in girls (Table 2). A positive and significant correlation (*r* = 0.651; *p* < 0.001) was found between the fat-free mass determined by anthropometry (27.8 ± 5.0 kg) and that determined by the creatinine content of 24-h urine (28.3 ± 5.5 kg) (Table 1). No significant differences were observed between these results, indicating that 24-h urine was collected appropriately.

**Table 2:** Twenty-four-hour urine data of the study population according to sex

	Total		Boys		Girls	
	Mean ± SD	Median (interquartile range)	Mean ± SD	Median (interquartile range)	Mean ± SD	Median (interquartile range)
Volume (mL/24 h)	870.4 ± 306.1	850.0 (650.0–1050.0)	891.8 ± 304.7	875.0 (650.0–1050.0)	848 ± 307.5	817.5 (636.3–1050.0)
24-h creatinine (mg/24 h)	718.59 ± 190.5	702.0 (584.0–851.0)	749.0 ± 188.0*	748.0 (627.0–873.0)	683.9 ± 188.2*	666.0 (551.0–816.5)
24-h sodium (mmol/24 h)	132.7 ± 51.4	128.0 (96.0–163.0)	141.8 ± 57.5*	136.0 (122.0–177.0)	122.4 ± 41.4*	119.5 (90.8–154.5)
24-h potassium (mmol/24 h)	49.4 ± 15.8	47.0 (39.0–59.0)	51.7 ± 17.0*	51.0 (40.0–63.0)	46.7 ± 13.8*	44.5 (37.0–55.3)
Sodium:potassium ratio (mmol/mmol)^a^	2.8 ± 1.2	2.6 (2.1–3.4)	2.9 ± 1.2	2.6 (2.2–3.7)	2.8 ± 1.2	2.5 (2.0–3.4)
Sodium:creatinine ratio (mmol/mg)	0.19 ± 0.06	0.18 (0.15–0.23)	0.19 ± 0.06	0.18 (0.15–0.23)	0.18 ± 0.05	0.18 (0.15–0.22)
Potassium:creatinine ratio (mmol/mg)^a^	0.07 ± 0.02	0.07 (0.06–0.08)	0.07 ± 0.02	0.07 (0.06–0.08)	0.07 ± 0.02	0.07 (0.06–0.08)

* *p* < 0.05 (differences between boys and girls)

^a^Variable not normally distributed

Assuming that the sodium eliminated in urine arose from the diet (132.7 ± 51.4 mmol/24 h) (Table 2), this excretion corresponded with a dietary salt intake of 7.8 ± 3.1 g/day in the entire sample, with differences according to sex (boys: 8.3 ± 3.4 g salt/day; girls: 7.2 ± 2.4 g salt/day; *p* < 0.05). According to WHO criteria [14], our results showed that 84.5 % of subjects aged ≤10 years had intakes of >4 g salt/day, and 66.7 % of those aged >10 years had intakes of >5 g salt/day.

By classification of our study cohort as normotensive and hypertensive children (12.7 % of children) [19], there were no differences in 24-h urinary excretion of sodium between normotensive (129.5 ± 48.6 mmol/24 h) and hypertensive children (155.5 ± 64.1 mmol/24 h; *p* > 0.05). However, SBP (*r* = 0.1574; *p* < 0.05) and DBP (*r* = 0.1400; *p* < 0.05) were correlated positively and significantly with 24-h excretion of sodium. Multiple regression analyses between 24-h urinary levels of sodium and SBP or DBP (including age, sex, and BMI as covariates) did not show a significant association, but when sex and BMI were eliminated, it was observed that SBP increased by 0.041 ± 0.020 mmHg (*R* ^2^ = 0.035; *p* < 0.05) and DBP by 0.041 ± 0.015 mmHg (*R* ^2^ = 0.035; *p* < 0.05) per each mmol/24-h urinary excretion of sodium.

Seventy-two percentage of children had a high urinary excretion of sodium (>100 mmol/24 h) [29]. Logistic regression analyses showed that the likelihood of excreting high levels of urine sodium increased by 15.9 % per each kg/m^2^ of BMI, after adjustment for sex (CI 1.041–1.290; *p* < 0.05). Overweight/obese subjects had higher urinary elimination of sodium (152.1 ± 59.2 mmol/24 h) than those of normal weight (124.6 ± 45.5 mmol/24 h; *p* < 0.01).

## Discussion

In our study, the mean intake of sodium was 2451 ± 558 mg/day. This result is similar to that reported in the enKid study [30], in which a sodium intake of 2200 and 2700 mg/day in boys aged 6–9 and 10–13 years, respectively, and 2000 and 2300 mg/day in girls aged 6–9 and 10–13 years, respectively, was noted. However, other authors have reported higher intakes. For example, Rodríguez-Artalejo et al. [31] recorded a sodium intake of 2700–3100 mg/day in Spanish subjects aged 6–7 years.

Bearing in mind that estimation of sodium intake is fraught with difficulties [7], and in agreement with numerous authors [7, 12, 32], the best way to determine salt intake is probably via the amount excreted in urine over a 24-h period.

Considering that the salt in urine arises from the diet, then 84.5 % of subjects aged ≤10 years had intakes of >4 g salt/day, and 66.7 % of those aged >10 years had intakes of >5 g salt/day [29]. These results are similar to those observed by Marrero et al. [33], who reported that 73 % of those aged 8–9 years and 73 % of those aged 13–17 years had a salt intake above the respective recommended maximum intake [29].

In Spain, only two studies have analyzed urinary excretion of sodium in children [16, 17], and those data are from 1985 to 2002. Luque-Otero et al. [16] reported a urinary excretion of sodium of 122.0 ± 40.0 and 127.0 ± 40.0 mmol/24 h in male individuals aged 8–9 years from Madrid and Santiago, respectively. Those results are slightly lower in comparison with that obtained in the male subjects of our study. Conversely, Maldonado-Martín et al. [17] reported a 24-h urinary excretion of sodium of 136.3 ± 63.3 mmol/24 h in subjects aged 6–14 years from Almería, which is similar to the value obtained in the present study. These results could be explained by the fact that, in the two last decades, dietary habits have changed dramatically toward an increased intake of processed food and restaurant/fast food, so salt intake would be higher than that in the 2000s [33–36]. In Danish adults, intake of household salt, estimated using a lithium marker, only contributed 8–10 % of total salt intake [37] indicating a high impact of other food sources, especially high processed food [38, 39].

Our value also resembles the 24-h urinary excretion of sodium of 132 ± 43 mmol/24 h reported by Cotter et al. [40] in a study of 139 Portuguese subjects aged 10–12 years; the 131 mmol/24 h observed by Shi et al. [41] observed in a group of 212 German boys aged 4–18 years; and the 129.2 ± 5.8 mmol/24 h observed by Marrero et al. [33] in a study of 103 British subjects aged 13–17 years. However, our results are higher than the 90.6 and 94.4 mmol/24 h found in a group of German males aged 7–10 and 10–13 years, respectively, and 80.6 and 95.0 mmol/24 h in a group of German females with the same ages, by Libuda et al. [42]; 97.5 and 89.0 mmol/24 h found in German boys and girls, respectively, aged 9–13 years reported by Alexy et al. [43]; 80.7 ± 3.4 mmol/24 h found by Marrero et al. [33] in 111 British subjects aged 8–9 years; 71 ± 23 mmol/24 h reported by Kritbjorndottir et al. [15] in 58 subjects aged 6 years from Iceland; 103 ± 43 mmol/24 h found by Grimes et al. [9] in 193 Australian subjects aged 5–13 years.

In the present study, 24-h urinary excretion of potassium was similar to that recorded by other authors [17, 41, 44]. Additionally, it was higher in comparison with that reported by Kritbjorndottir et al. [15], who found a 24-h excretion of potassium of 31 ± 11 mmol/24 h.

We found differences according to sex in urinary excretion of sodium (Table 2), having observed that boys had higher excretion than girls [7, 17, 33, 40, 43–47]. These differences could be because of their overall higher food intakes and differences in food habits [8]. Indeed, we found that boys had higher energy intakes (2140 ± 299.3 kcal/day) and food consumptions (1977 ± 479.8 g/day) than girls (2049 ± 331.1 kcal/day and 1842 ± 406.6 g/day; *p* < 0.05 in both cases).

Several studies have examined the consequences of a high dietary intake of salt on BP. A meta-analysis of 10 controlled trials showed that dietary intake of salt is related to higher BP, and that a reduction in salt intake by 42 % in children resulted in a fall in SBP and DBP of 1.17 and 1.29 mmHg respectively [48]. In our study, 12.7 % of children were classified as “hypertensive” and we found that, for each mmol/24 h of sodium excreted in urine, the SBP and DBP increased by 0.041 and 0.043 mmHg, respectively. These effects might seem small, but an increase/reduction in BP during childhood can have important consequences [33, 49], and a reduction in salt intake could lead to other positive health-related outcomes [33, 50].

Children who were overweight or obese had higher urinary excretion of sodium than those with normal weight. These findings have been observed in other studies carried out with young people and adults [4, 39, 51–53]. In particular, our results are in agreement with the results shown by Libuda et al. [42], who found a positive trend between urinary excretion of sodium and increase in body weight and %BF among 364 children and adolescents. Hulthén et al. [39] observed rising values for body weight and the BMI in Swedish subjects aged 18–20 years across quartiles of sodium excretion. This phenomenon could be because a high intake of salt stimulates the appetite and thirst, thereby increasing the total intake of energy [39, 54]. However, in the Dortmund Nutritional and Anthropometric Longitudinally Designed (DONALD) study [42], inclusion of sugar-sweetened soft drinks and total energy intake in the study design did not elicit this association, suggesting that other mechanisms may be operating. In a study with rats, Fonseca-Alaniz et al. [55] suggested that high consumption of salt contributes to an increase in adiposity because of an increase in the capacity to incorporate glucose into lipids. They also found that a higher activity of lipogenic enzymes may promote adipocyte hypertrophy and excessive accumulation of fat.

### Strengths and limitations of the study

One of the strengths of the present study was the use of an objective indicator, 24-h urinary sodium, to measure total salt intake. In addition, we used a validated protocol for 24-h urine collection. We had only one 24-h urine excretion per child, which might be considered a limitation. Other limitations are that our sample was not representative and was relatively small (*n* = 205).

## Conclusions

Sodium intake, as estimated by 24-h urinary excretion, was (on average) higher than that recommended. Reducing the sodium content children’s diets is a sound policy to reduce cardiovascular risk.
